# Tongue position and paharyngeal airway dimension with mandibular setback procedure among skeletal class III cases

**DOI:** 10.6026/973206300212815

**Published:** 2025-08-31

**Authors:** Vyshak T.V., Sanghamitra Jena, Nivedita Sahoo, Biswaroop Mohanty, Bhagabati Dash

**Affiliations:** 1 Department of Orthodontics and Dentofacial Orthopedics, Kalinga Institute of Dental Sciences, KIIT (Deemed to be University), Bhubaneswar, Odisha, India

**Keywords:** Tongue position, paharyngeal airway dimension, mandibular setback, skeletal class

## Abstract

Mandibular setback surgery is frequently used to address this condition, enhancing both facial aesthetics and bite function. Therefore, it is of interest to assess the dimensions of the pharyngeal airway which has undergone change in the position of tongue as a consequence of setback surgery of the mandible. This study found significant reductions in airway dimensions and posterior tongue displacement following mandibular setback surgery. There is reduction in the dimensions of the oropharyngeal airway.

## Background:

Skeletal Class III malocclusion, mark by an overly prominent lower jaw, which affects about 5-15% of people globally. Mandibular setback surgery is frequently used to address this condition, enhancing both facial aesthetics and bite function [1]. However, concerns persist regarding its impact on upper airway dimensions and tongue placement, which could contribute to respiratory issues like obstructive sleep apnea [2]. Recent research has explored how mandibular setback surgery influences tongue positioning and pharyngeal airway dimensions, but findings remain inconsistent. Gaining a thorough understanding of these effects is essential for improving outcomes, minimizing risks and ensuring patient well-being [3]. The integration of surgical and orthodontic techniques for severe mandibular prognathism has enabled patients who were previously considered permanently impaired to achieve normal function and satisfactory aesthetics. Since the 1950s, analyzing facial soft tissues has played a vital role in orthodontic treatment planning. Today, orthognathic surgery is commonly used to optimize both function and facial appearance, making the behavior of soft tissue structures post-surgery a key focus of interest [4].

Correcting Class III dentofacial deformities can involve maxillary advancement, mandibular setback, or bimaxillary procedures. Determining the most suitable approach is sometimes challenging [5]. While all methods are typically effective in resolving dental malocclusions, their impact on appearance varies, with only one yielding the most aesthetically pleasing profile. Though studies of post-surgery stability often examine hard tissue positions, some have assessed the correlation between hard and soft tissue changes [6]. Various methods for evaluating upper airway dimensions include; CT, MRI and Lateral Cephalograms. These techniques face limitations, such as increased radiation exposure and high costs. Acoustic Pharyngometry (AP), a newer method, is a non-invasive, chair-side technique based on acoustic reflection, offering a practical alternative for routine clinical application [7, 8, 9-10]. Therefore, it is of interest to examine the relationship between the extent of mandibular setback and changes in tongue position, to measure alterations in pharyngeal airway dimensions following surgery.

## Materials and Methods:

As a framework for this systematic review the PRISMA checklist was utilized Protocol and Registration: The PROSPERO ID for this systematic review has been registered under the following ID- CRD42024506268.The PICO format was employed to define the eligibility criteria for the above study which includes; Population: All Individuals having features of Angle's Class III malocclusion along with mandibular prognathism or extreme deformity of skeleton who have been diagnosed for mandibular setback surgery, Intervention: Mandibular setback surgery.

## Comparison:

Parameters measured before and after surgery.

## Outcome:

Change in dimensions of the pharyngeal airway space and position of tongue pre and post mandibular setback surgery. In order to undertake this systematic review we followed the following:

## Inclusion criteria's:

Randomized clinical trials, Interventional studies, Comparative studies, Patients diagnosed with Angle's Class III malocclussion, Patients undergoing setback surgery of mandible exclusively, and Studies with a minimum follow-up period of two years. Studies with Literature reviews descriptive studies, Individual case reports or case series, Studies not published in English, Patients with syndromes, systemic diseases, or cleft conditions and existing systematic reviews were excluded. A systematic literature review was planned to search in several electronic databases -Web of Science (core database), ProQuest, Scopus, Science Direct and PubMed. A thorough Search was conducted from the inception of the database till 2023 November. The search strategy was limited to English language publications. The MeSH (Medical Subject Headings) terms were combined using Boolean operators "AND" and "OR" to construct search keywords for the database. Figure 1 (see PDF) indicates the PRISMA flow chart. The obtained data was statistically evaluated.

## Results:

The data required for review were selected in two steps. During first round, only those articles whose titles and abstracts were tallied with the review topic were included. This resulted in procurement of two hundred and three articles out of which the number of duplicates found were sixty one. For the next step we had hundred and thirty nine articles to which the inclusion and exclusion criteria were applied. The end result of the above resulted in 20 articles. Out of the 20 articles 3 were excluded as they were not reported in English, four articles were irrelevant. At the end we had a sum of 13 articles on which we conducted our systematic review and meta-analysis (Table 1 - see PDF).

## Green circles:

Represent questions that were met (low risk of bias).

## Red circles:

Represent questions that were not met or were unclear (high or unclear risk of bias.

## Risk of bias:

In order to eliminate any kind of bias we assessed each study individually which has been tabulated in Figure 2 and 3 (see PDF). The NIH Quality Assessment Tool for non-randomized studies is commonly used to evaluate the methodological quality of studies based on various domains such as participant selection, outcome assessment, and statistical analysis.

## Criteria used in the NIH tool:

[1] Was the study design appropriate for the question in review?

[2] Was the study subject's representative of the general population?

[3] Were there clear inclusion and exclusion criteria?

[4] Were the outcome measures valid and reliable?

[5] Was there adequate statistical analysis?

[6] Was there loss to follow-up or incomplete outcome data?

[7] Was the study controlled for confounding factors?

## Pharyngeal Airway Dimension:

The meta-analysis of 10 studies revealed a significant reduction in the dimensions of pharyngeal airway postmandibular setback surgery. The pooled mean difference was -2.34 mm (95% CI: -3.53 to -1.15, p < 0.001).

## Forest plot:

After meta-analysis a forest plot was plotted illustrating the results of the same (Figure 4 - see PDF)

## Tongue position:

The meta-analysis of 8 studies revealed a significant posterior displacement of the tongue after mandibular setback surgery. The pooled mean difference was -1.87 mm (95% CI: -2.93 to -0.81, p < 0.001).

## Forest plot:

After meta-analysis a forest plot was plotted illustrating the results of the same (Figure 5 - see PDF).

## Heterogeneity and publication bias:

In order to assess heterogeneity the I^2 statistic was used. The results showed moderate to high heterogeneity for both pharyngeal airway dimension (I^2 = 63.2%) and tongue position (I^2 = 57.1%). Funnel plots were used to assess publication bias, and the results showed no significant asymmetry.

## Sensitivity analysis:

In order to asses' robustness of the results sensitivity analysis was performed. The analysis showed that the results were not significantly affected by the exclusion of any single study.

## Subgroup analysis:

Subgroup analysis was performed to explore the effects of different surgical techniques and follow-up periods on the outcomes. The results showed that the effects of mandibular setback surgery on pharyngeal airway dimension and tongue position were not significantly different between different surgical techniques or follow-up periods.

## Discussion:

Mandibular setback surgery has a profound effect not only on the muscles of the tongue but also position of the hyoid bone, potentially reducing the pharyngeal airway space (PAS). Deegan in 1995 observed that the dimensions of the pharyngeal airway space have a significant impact on oropharyngeal muscle function. If the oropharynx muscle fails to counteract pressure, airway collapse may occur. Research has shown that obstructive sleep apnea reduces the activity of various respiratory muscles during sleep, including the genioglossus and tensor palatini [11, 12]. Adult class III patients with a substantial negative overjet will inevitably require both orthodontics and orthodontic surgery. The skeletal, soft tissue and dental spatial and dimensional relationship in the oropharyngeal area may be altered, even though this might significantly improve the patient's facial profile, smile aesthetics, and self-esteem. The pharyngeal airway dimensions may be impacted by the changes in tongue height and length after mandibular setback (MS) surgery. CBCT and lateral cephalograms aid in assessing the size of the airways. There is little research linking changes in the dimensions of the tongue to changes in the volumetric and linear airways [13].

Sahoo *et al.* concluded forms his study that, the appraisal of tongue length and height after MS surgery should be an integral part of diagnosis and treatment planning. The retro-positioning of tongue and increase in its height after MS surgery may compromise pharyngeal airway especially PAS [13]. Research has provided varied outcome on changes in the oropharyngeal airway by different authors. Tselnik and Pogrel noted a 6.1% increase immediately after surgery, while other studies observed significant decrease over time. The lack of randomized controlled trials limited this review's ability to compare surgical outcomes with a control group, as lower pharyngeal depth generally stabilizes early in life [7]. The systematic review included 22 studies of moderate quality, with 13 included in the meta-analysis. Most studies used two-dimensional cephalometric radiography, which, while useful, does not capture three-dimensional changes. Although CT scans offer greater precision, few studies have used this method for assessing volumetric changes following surgery. It has been reported that, posterior positioning of the tongue and hyoid bone after mandibular setback (MS) surgery which may have a negative impact on upper airway and may lead to breathing disorders such as obstructive sleep apnea (OSA) [7, 14]. The results of the study have revealed that there is definite reduction in the dimensions of the oropharyngeal airway and there is substantial evidence to prove that there is a change in position of tongue potentially impacting the respiratory function and quality of sleep. However further research is required to augment the results of the present study.

## Conclusion:

We show significant reductions in airway dimensions and posterior tongue displacement following mandibular setback surgery. However, variability in measurement methods, follow-up durations, and control over head and neck positioning limited the analysis. Changes in head position, as described by Hellsing, or inconsistent tongue positioning during imaging could influence results.
